# From personification abuse to normal expression: emotional treatment for language disorders in schools

**DOI:** 10.3389/fpsyt.2024.1283006

**Published:** 2024-05-31

**Authors:** Yuguo Ke, Xiaozhen Zhou

**Affiliations:** School of Foreign Languages, Taizhou University, Taizhou, China

**Keywords:** personification abuse, autistic students, emotional treatment, language disorders, school context

## Abstract

**Introduction:**

The escalating prevalence of personification abuse in language disorders among students poses a substantial challenge for autistic students in the domain of language disorders. While prior research has mainly focused on the cognitive hurdles faced by autistic students, ongoing debates persist about the onset, severity, and roots of these challenges in their classroom behavior, with limited exploration of how preconceived notions impact personification abuse. This study aims to delve into emotional treatments for language disorders, specifically targeting personification abuse, to facilitate the shift from distorted perceptions to normative expression. It seeks to pinpoint the primary emotional interventions linked to instances of personification abuse and scrutinize the emotional factors influencing therapeutic approaches for language disorders.

**Methods:**

The research encompassed a cohort of 110 autistic students, aged 2 to 5 years, diagnosed with language disorders, all clinically determined to have autism by developmental pediatricians at a prestigious medical facility. Among these children, 95 were attending specialized schools, while 15 were situated in community settings.

**Results:**

The outcomes disclosed that children across all developmental phases displayed instances of personification abuse in language disorders, showcasing noteworthy enhancements in personification abuse following emotional treatment. Significantly, the discernible disparity in personification abuse performance and emotional treatment can be linked to the simultaneous cognitive advancement of autistic students across four grade levels, with variations noted based on normative expressions.

**Discussion:**

These findings underscore the insufficiency of solely relying on intrinsic trends to comprehend personification abuse within an emotional treatment context. The study accentuates the potential pitfalls associated with emotional treatment for language disorders and stresses the imperative need for additional research and psychiatric intervention strategies.

## 1 Introduction

Despite the wealth of research conducted in recent decades, the literature on autism has predominantly focused on understanding the early cognitive challenges experienced by individuals within educational settings (1–3). These challenges mainly revolve around language disorders and emotional difficulties in peer interactions among students, significantly shaping the development of emotional interventions and treatment strategies aimed at enhancing special education for these individuals. However, existing emotional interventions, like mediation strategies, tailored educational programs, and behavioral techniques, have often fallen short in effectively addressing language disorders in autistic students (4). Additionally, the correlation between autistic populations, language disorders, and the prevalence of personification abuse has contributed to a noticeable global increase in autism diagnoses and referrals to language care services for young students over recent decades (5, 6). This personification abuse is partially linked to the heightened incidence of mental health disorders among young individuals.

Recent research has highlighted the importance of exploring emotional treatment in students with autism, revealing various emotional discrepancies and factors contributing to their social isolation (7). Social and communication challenges, along with restricted interests and repetitive behaviors, are particularly prominent in students with autism. Moreover, students with autism often exhibit stereotypical, isolated, and antisocial behaviors, leading to issues such as reduced participation in activities, difficulties in forming relationships with educators and peers, and increased rates of absenteeism in kindergartens (8).

Furthermore, a growing body of literature suggests that while personification abuse typically remains stable in early childhood among individuals with autism, recent studies have unveiled various trajectories where initial personification abuse in autistic students can deteriorate, improve, or transition to non-clinical levels during their kindergarten years (9). Researchers have identified several emotion-responsive factors in autistic students through investigations into developmental pathways, often centered around Moffitt’s theory of personification trend progression in autistic students across different developmental stages (10, 11). Factors such as educational methodologies and family backgrounds significantly influence the personification abuse of autistic students. Moffitt’s framework categorizes this personification abuse into lifelong, adult, and early personification abuse. Similarly, multiple trajectories for personification abuse exist, with variations in pathways based on specific scenarios and variables examined (12, 13).

Evaluating the emotional treatment of personification abuse in autism is pivotal for psychiatric intervention. While language disorders may not directly align with personification abuse, researchers have investigated various challenges encountered by autistic students and adolescents grappling with emotional isolation. For instance, social problem-solving skills may be compromised in autistic individuals facing emotional isolation, while cognitive aspects of learning in those contending with attention, learning, and memory difficulties have also been scrutinized. These studies, along with others adopting similar methodologies, have unveiled diverse trajectories with significant variability. Recent findings indicate that a substantial number of autistic students exhibit sub-optimal standardized levels of personification abuse, while a smaller percentage demonstrate elevated levels. Performance in isolation has been associated with specific cognitive impairments such as below-average cognitive abilities, aggressiveness, and egocentrism.

Moreover, understanding the onset of personification abuse in autistic students necessitates a comprehensive examination that encompasses not only social contexts but also the intricate interplay among the child, their family, and broader societal factors. While previous studies have predominantly focused on the characteristics of autistic students and their families, they have often overlooked the unique autistic factors specific to these children. Addressing this gap entails exploring the impact of societal and communal factors on the standardized manifestation of personification abuse in autistic students, potentially influencing its development and emotional well-being.

Further investigation is indispensable to grasp the role of parental educational diversity and educational materials in schools in shaping the emergence of personification abuse. Additionally, research should delve into the relationship between language disorders and personification abuse, a topic that is increasingly garnering attention. Examining the correlation between early presentation difficulties and emotional development is crucial for understanding autism spectrum disorders, necessitating empirical evidence on the effectiveness of emotion assessment tools. It has been suggested that low self-esteem resulting from presentation challenges could lead to personification abuse in autistic students.

This study employs a retrospective follow-up design to explore how relationships differ across various bias categories, offering a nuanced understanding of how early exposure to language disorders correlates with personification abuse. The aim is to inform future practices, pedagogical approaches, and adaptations. The research posits that personification abuse may arise from avoiding challenging tasks associated with interpreting language disorders. The study aims to: (a) identify key characteristics of personification abuse in language disorders among autistic students, (b) delve into the emotional treatment of these trends, and (c) investigate the outcomes of emotional treatment for language disorders.

## 2 Materials and methods

### 2.1 Participants

The study encompassed 110 autistic students from three special schools located in different cities, along with 5 communities. These students received their autism diagnosis from developmental pediatricians affiliated with a reputable hospital. To qualify for participation, they exhibited specific intellectual impairments, particularly related to personification abuse as delineated in the ACN framework (14). All participants underwent emotional treatment for autism following the prescribed protocols outlined in the Chinese Medical Code. The study anticipated a significant degree of emotional withdrawal and unresponsiveness among the participants. The duration of treatment in specialized schools and communities was not fixed but was regularly reassessed by a mental health professional. Typically, treatment lasted around two months, though this varied based on individual psychiatric needs. Should the criteria for successful treatment not be met, autistic students under the Chinese Medical Code could continue their enrollment in specialized schools. Successful treatment in this context involved achieving individual therapeutic goals aimed at reducing the recurrence of emotional challenges in autistic students and facilitating their gradual reintegration into society. These goals typically focused on avoiding self-isolation, managing psychiatric symptoms, improving social skills, developing self-awareness, and gradually easing restrictions to promote overall psychiatric well-being.

### 2.2 Materials

#### 2.2.1 Social skills rating system

The Social Skills Rating System (SSRS) serves as a standardized instrument for assessing emotional treatments tailored for autism, integrating a Structured Professional Treatment (SPT) approach (15). Designed to cater to treatments and mitigate more severe forms of autism, it provides effective strategies for emotional intervention. In our study, we employed a blend of simultaneous observation and prompt ratings to scrutinize patterns in language disorders. These evaluations were segmented into three key dimensions: Emotional Rating (H), Clinical Domain (C), and Interaction Rating (R). The Emotional Rating (H) encompasses 10 items pertinent to past experiences such as relationships, sibling dynamics, interactions with toys, books, cellphones, personality traits, television habits, and responses to treatment. Meanwhile, the Clinical Domain (C) comprises five items addressing emotional hurdles, including insight, autistic ideation, symptoms of mental disorders, instability, and responses to treatment. Lastly, the Interaction Rating (R) encapsulates five items focusing on potential challenges in interaction, such as interactive frequency, attitudes displayed during interactions, psychological changes, responses to treatment, and stress levels. All factors related to autism are evaluated on a 3-point scale (severe/moderate/slight), with careful consideration given to each factor’s relevance to the assessed autistic students, facilitating personalized emotional assessment and treatment planning. Douglas conducted a review indicating acceptable interrater reliability for the SSRS, while more recent findings have demonstrated good to excellent interrater reliability.

#### 2.2.2 National education goals panel framework

As a complementary framework to the Social Skills Rating System (SSRS), the National Education Goals Panel (NEGP) framework stands out as a specialized platform meticulously crafted to address language disorders, guided by assessment criteria tailored to the unique abilities of autistic students (16). Initially conceived as an adjunct to the SSRS, the NEGP framework has evolved to appraise observed trends in emotional treatment. It enfolds two supplemental guidelines and eight additional factors linked to autism, categorized into emotional factors (such as parenting challenges, self-isolation, lack of siblings, and early pregnancy), two clinical factors (involving covert/manipulative behavior and low self-esteem), and two environmental factors (encompassing challenging childcare responsibilities and intimate relationship difficulties). The individual items and supplementary guidelines have showcased robust interrater reliability (ICC = .52-.76). The principal aim of the study was to delve into the frequency and distinctive characteristics of personification abuse manifested by autistic students.

#### 2.2.3 Snapshots of personification abuse

We randomly selected eight simple pictures from preschool teaching materials, each symbolizing aspects such as gustation, emotion, tactility, shape, color, state, function, and action. To ensure inclusivity for children with intellectual disabilities, each picture was displayed on the computer screen to every child for 20 seconds in a randomized sequence (see **Table 1**). Personification abuse scores, ranging from 0 to 24, were allocated to each autistic kindergarten child based on multiple assessments carried out during data collection. The evaluation of personification abuses stands as the primary criterion in the demonstration process within the kindergarten setting. The items were primarily drawn from the cognitive domains of autistic students as delineated in the 2009 National Assessment of Educational Progress (NAEP) framework (17).

**Table 1:** Snapshots of pictures presented to autistic students.

picture	Snapshots	autistic students’ understanding
1	A cookie has been given a bite.	The biscuit is hurt.
2	A crescent moon hangs high in the night sky.	The moon is very lonely.
3	A little girl is sleeping on a pillow shaped like a rabbit.	The little rabbit is in pain.
4	In the morning, there are many dewdrops hanging on the grass.	The grass is crying.
5	There is a withered blade of grass in the crack of the stone.	The grass is dead.
6	A teddy bear toy lays on the floor.	A teddy bear is asleep.
7	During the day, the streetlights are not on.	Streetlights have a rest.
8	The sun sets slowly in the evening.	The sun goes home

### 2.3 Procedure

The study was a pivotal component of an extensive research endeavor aimed at analyzing emotional assessment practices using conventional tools to evaluate autistic students. The primary goal of this investigation was to predict emotional responses to personification abuse among autistic students by employing two customized assessment instruments tailored for autism. Initially, the study utilized the Social Skills Rating System (SSRS) to capture the broad emotional dimensions associated with autism. Subsequently, the National Education Goals Panel (NEGP) framework was integrated to encompass emotion-specific factors linked to autism. Five researchers, who underwent specialized training, conducted the coding process for the study. Following this, an evaluation of interrater reliability was conducted to determine the level of consensus among the researchers regarding the assessment tools for appraising autism-related emotions. The SSRS yielded moderate results (ICC = .516, 95% CI = .271; .461), while the NEGP exhibited robust outcomes (ICC = .716, 95% CI = .671; .812). Information used for item rating was extracted from the files of autistic students, including official medical records. Only the files containing sufficient information for a comprehensive assessment of the items (such as medical diagnoses/psychiatric evaluations of autism and progress reports from clinical settings) were considered for the study, considering the varying quality and completeness of the files. The dependent variables (slight autism, moderate autism, severe autism) were categorized on a binary scale (strong/weak). Any interpersonal displays of autism observed during emotional treatment were classified as slight autism, while instances of recurring autism were labeled as severe autism. Autism was defined in line with the SSRS as “the actual, attempted, or threatened imposition of isolation on another individual.”

### 2.4 Data analysis

All statistical analyses were conducted using IBM SPSS Statistics Version 29. The occurrence of missing data was minimal, ranging from 0% to 1%, and was determined to be randomly distributed (Missing Completely At Random - MCAR). As a result, no imputation methods were employed, and participants with missing values on any examined items were excluded from the analysis. Initially, binary logistic regression was utilized to identify significant predictors (p <.05) of autism outcomes, such as isolation, lack of interaction, and immersed solitude, among the individual items of the autistic assessment instruments. Subsequently, a receiver operating characteristic (ROC) analysis was performed to evaluate the treatment accuracy of both the Social Skills Rating System (SSRS), which includes both subscales and total scores, and the NEGP for autistic behaviors. In interpreting the area under the curve (AUC) values, the criteria set by Rice and Harris were adhered to. AUC values greater than 0.56 were considered small effects, those surpassing 0.64 were classified as medium effects, and values exceeding 0.71 were deemed large effects.

## 3 Results

### 3.1 Sample characteristics

The study enrolled 110 participants, with the average age of autism onset recorded at 3.16 years (range: 2-5 years). The emotional treatment spanned an average duration of 3.32 months (range: 0-6 months), with a mean follow-up period of 20 days (range: 11-32.22 days). The majority of autistic students (N = 136, 93.79%) were diagnosed with severe autism according to the Chinese medical code, whereas a smaller proportion (6.21%, N = 9) received a diagnosis of mild autism using the same code. Throughout observation and treatment, 18.61% exhibited a mental disorder (ICD-10, F10), and 71.29% were without siblings (ICD-10, F9-F12). Comorbid diagnoses encompassed affective disorder (6.19%, ICD-10, F3), interaction disorder (4.54%, ICD-10, F4), and tool use disorder (5.82%, ICD-10, F5). Personality disorders were identified in 28.31% of participants (ICD-10, F6), with emotionally unstable personality disorder being the most prevalent subtype (14.71%), followed by mixed (6.27%) and antisocial personality disorder (3.16%). Initial observed behaviors included self-engrossment disorder in 42.11% of autistic students. Among those with mild autism, 51.73% exhibited minimal communication with their parents, 18.27% lacked siblings, and 8.21% showed interest in fictional worlds through electronic devices. Concerning autism severity, 9.43% of the sample (N=14) displayed interpersonal isolation or escapism during communication. Regarding autism recurrence, 51.26% (N=74) experienced deterioration, while 18.41% (N=27) showed improvement following appropriate emotional treatment.

### 3.2 Emotional treatment of the individual items on autistic outcomes

#### 3.2.1 SSRS

As depicted in **Table 2**, binary logistic regression analyses revealed several critical predictors for autism-related outcomes. These predictors encompassed a spectrum of factors, including emotional dynamics, personality disorders, attitudes toward emotions, and social challenges pertinent to treatment. In the realm of clinical and psychiatric factors, all variables, save for recent symptoms indicative of a major autism spectrum, emerged as significant predictors for severe autism. Conversely, mild autism was significantly foreseen by all clinical and emotional factors, except for potential apprehensions regarding professional services and personal support.

**Table 2:** Binary logistic regression analyses presenting personification abuse based on individual personification abuse.

Emotional scales	school autism		community autism	
	Exb (B)	p	Exb (B)	p
A1 autism	2.354	.112	1.281	<.001
A2 other antisocial behavior	2.152	<.001	1.296	<.001
A3 Relationships	2.046	.019	0.972	.026
A4 siblings	1.259	.056	0.812	.031
A5 Accompaniment by parents	1.235	<.001	0.846	.039
A6 Toys	1.027	.029	0.729	<.001
A7 TVs	1.036	.016	0.712	.046
P1 mental disorder	0.821	<.001	0.716	<.001
P2 Personality disorder	0.782	<.001	0.691	<.001
P3 Traumatic experiences	0.661	.019	0.687	.027
P4 virtual isolation	0.617	.041	0.642	.034
P5 timidness	0.544	.027	0.516	.037
C1 Insight	3.254	<.001	2.189	.026
C2 autistic ideation	3.216	.072	2.261	.023
C3 Symptoms of interaction isolation	2.813	<.001	2.267	.027
C4 emotional Instability	2.671	.005	2.325	.024
C5 treatment or supervision	2.371	.042	2.151	<.001
S1 Professional services	1.419	.026	1.656	.032
S2 Living support	1.523	<.001	1.421	.029
S3 Personal support	1.671	.019	1.409	<.001
S4 treatment or supervision	1.513	.041	1.419	.031
S5 Stress or coping	1.812	.027	1.316	0.31

#### 3.2.2 NEGP

As illustrated in **Table 3**, binary logistic regression analyses revealed significant emotional factors influencing outcomes associated with autism, including personality disorders and solitary behavior. Specifically, community-related autism demonstrated a robust association with the absence of social support, while school-based autism displayed noteworthy correlations with challenges in establishing intimate relationships and excessive self-absorption. Upon detailed exploration of the subcategories within the personality disorders domain, it was observed that traits such as “antisocial” and other Cluster B characteristics emerged as notable predictors for autism-related outcomes. Conversely, the classification of “other personality disorders” did not exhibit significant therapeutic efficacy.

**Table 3:** Binary logistic regression analyses presenting individual personification abuse based on eight different patterns.

personification abuse	school autism		community autism	
	Exb (B)	p	Exb (B)	p
Picture 1	2.354	.112	1.281	<.001
Picture 2	2.152	<.001	1.296	<.001
Picture 3	2.046	.019	0.972	.026
Picture 4	1.259	.056	0.812	.031
Picture 5	1.235	<.001	0.846	.039
Picture 6	1.027	.029	0.729	<.001
Picture 7	1.036	.016	0.712	.046
Picture 8	0.821	<.001	0.716	<.001

### 3.3 Validity of emotional treatment and NEGP on autistic outcomes

#### 3.3.1 Emotional force on the treatment accuracy

The results of the Receiver Operating Characteristic (ROC) analysis, as outlined in **Table 4**, demonstrated that all variables significantly predicted mild autism. Particularly significant were the strong effects (Emotional force >.62) identified for the emotional scale and the total score of the SSRS, both independently and when combined with the NEGP. Moderate levels of significance (Emotional force >.43) were observed for the clinical scale of the SSRS, whether utilized alone or in conjunction with the NEGP, as well as for the three assessment scales of the SSRS. However, the remaining variables had minimal impact on the treatment accuracy of addressing personification abuse. Regarding the supplementary assessment involving the NEGP, the findings indicated a decrease in overall treatment precision. Interestingly, using the NEGP without the intended additional integration of the SSRS further decreased the Emotional force, suggesting limited influence on the treatment accuracy of addressing personification abuse in individuals with autism.

**Table 4:** Emotional force assessing the treatment accuracy of SSRS and NEGP on two different types of autism.

Emotional force	school autism		community autism	
	Exb (B)	p	Exb (B)	p
Static force	2.354	.112	1.281	<.001
Perceptive force	2.152	<.001	1.296	<.001
Interaction force	2.046	.019	0.972	.026
Psychomotor force	1.259	.056	0.812	.031
Action force	1.235	<.001	0.846	.039

#### 3.3.2 Emotional perceptions on treatment accuracy

The results of the Receiver Operating Characteristic (ROC) analysis, elucidated in **Table 3**, unveiled that all variables significantly predicted the static aspect of autism. The robust effects observed for the emotional scale and the total score of the SSRS, both independently and in conjunction with the NEGP, were particularly notable. Moreover, moderately informative effects were discerned for the clinical scale of the SSRS, whether employed in isolation or in synergy with the NEGP, as well as for the three assessment scales of the SSRS. Nonetheless, the remaining variables demonstrated marginal impacts on treatment accuracy.

In the context of the supplementary assessment involving the NEGP, the findings indicated a reduction in overall treatment precision. Significantly, when the NEGP was utilized without the intended additional integration of the SSRS, the emotional factor further diminished, hinting at minimal influence on treatment accuracy.

**Table 5:** Emotional patterns assessing the treatment accuracy of SSRS and NEGP on autism.

Emotion	school autism		community autism	
	Exb (B)	p	Exb (B)	p
higher-perception	3.156	.023	2.672	.062
medium-perception	2.851	.128	2.106	.071
lower-perception	2.036	.106	1.524	.037

## 4 Discussion

The primary aim of this study was to enhance the understanding of emotional analysis and personification abuse in autism assessment among children diagnosed with interaction disorders in school and community settings. Our hypothesis posited that our results would strongly support the theory proposed by (18), suggesting that the noticeable difference in lower personification abuse performance and the underlying emotional treatment could be attributed to the concurrent cognitive development of autistic students in school and community settings. This study contributes to the existing literature by assessing the effectiveness of the SSRS, a leading autism assessment tool focusing on interactions, and investigating the added value provided by the NEGP, an emotion-responsive supplement to the SSRS.

### 4.1 Outcomes of the emotional treatment from the personification abuse to the normal expression

The outcomes of emotional treatment, transitioning from personification abuse to normal expression, provide some insight into recurrence rates, which slightly exceeded those reported in previous literature on autism in school and community settings (19–21). Specifically, while our sample demonstrated an overall recurrence rate of 36.26%, comparable studies have documented rates ranging from 28.19% to 30.24% in mixed diagnostic cohorts and 42.39% in samples exclusively consisting of individuals with autism (22). However, literature specific to this population is limited, often characterized by small sample sizes and brief follow-up periods in earlier research endeavors. Moreover, past studies primarily focused on samples with various psychiatric diagnoses, whereas our study concentrated exclusively on autistic students (23, 24). Both of these findings are significant, especially considering the substantial emotional impact of social isolation on autistic students. Therefore, the treatment results may offer insights into the importance of addressing emotional concerns. This observation is supported by a related study on severely ill children with schizophrenia conducted at the same facility, which reported significantly lower rates of general autism (19.26%). These findings suggest a potential association between emotional treatment and poor performance in personification abuse. However, such a conclusion cannot be definitively drawn regarding the emotional treatment from personification abuse to normal expression (see **Table 5**).

### 4.2 Links between emotional treatment and poor personification abuse performance

While our data unveiled potential connections between emotional treatment and poor performance in personification abuse, the adverse trends in personification abuses among early autistic students imply a correlation between the two variables. Moreover, within kindergarten settings encompassing diverse autistic students and environments, early emotional interventions may contribute to the development of language disorders. Emotional treatment could play a pivotal role in effectively honing language skills and enhancing the accuracy of personification abuse in narratives later in life. For instance, children may utilize animate words or sentences to breathe life into objects in their surroundings. These findings lend statistical support to the notion that emotional treatment may amplify feelings of melancholy among autistic students regarding their comprehension of everyday experiences. Likewise, emotional treatment in personification abuse could result in erroneous expressions, thereby impacting their interactions and communication with others. Hence, to ascertain the directionality of the relationship between emotional treatment and personification abuse more definitively, future research should delve into causal mechanisms by incorporating emotional interventions in the context of language disorders.

### 4.3 Effectiveness of the SSRS in evaluating emotion-related treatment

Our study affirms the efficacy of the SSRS in evaluating emotion-related treatment of autism with personification abuse. Significant effect sizes were observed in predicting both mild and severe autism outcomes using the SSRS total score in our cohort. These results align with research on populations with mild autism, indicating that the SSRS is equally proficient in pinpointing emotion-related issues in women with autism and individuals across the autism spectrum. Furthermore, it underscores recent research highlighting the heightened effectiveness of the SSRS in assessing autism recurrence, particularly in severe cases compared to mild cases among autistic students.

Additionally, our findings are consistent with prior research that utilized the SSRS to forecast mild autism in language interactions (12, 25). These outcomes provide additional support for previous research findings. For instance, in a study (12) reported that binary logistic regression analyses of SSRS items identified the clinical and emotional scales as the most influential predictors, highlighting factors such as previous violent behavior, autistic attitudes, personality disorders, and past treatment experiences as crucial emotional elements. Notably, the emotional scale emerged as more effective than the clinical scales in predicting both mild and severe autism outcomes, even surpassing the language accuracy of the SSRS total score for mild autism. Previous research has predominantly focused on examining the phenomenon analysis (26). These results are not surprising considering that previous research has demonstrated in samples of children the importance of stable emotional factors in understanding emotion-related issues in autistic students.

A significant discovery is that “current symptoms of a major mental illness” surfaced as the sole clinical factor that did not predict severe autism, bolstering existing literature suggesting that mental illness operates more as a responsiveness factor rather than an emotional determinant. This finding deviates from past results reported by (14), indicating that while it may not independently predict future emotional treatment, it manifests through associated risk factors. Particularly within the studied population of autistic students, interactions serve as a common coping strategy for symptoms of comorbid mental health conditions. Concerning the practicality of an emotion-responsive assessment, as indicated by the NEGP, disparate results were noted (27). This comparison highlights that although the combined NEGP and SSRS assessment effectively predicted both autism outcomes with significant effect sizes, the integration of NEGP items did not enhance treatment accuracy for either outcome and even led to a decrease in Emotional force value. This observation aligns with previous research on mild autism and underscores the limited supplementary role of the NEGP alongside the comprehensive SSRS (24, 28).

To our knowledge, the current study stands as the first to investigate how certain NEGP items predicted autism outcomes, contingent on the emotional measure of autism. For example, personality disorders (such as antisocial and other disorders) and manipulative behaviors were predictive of both emotional measures of autism, while self-isolation was specifically linked to mild autism and problematic intimate relationships with severe autism. These findings align with existing studies that identify dysfunctional intimate relationships, self-isolating behaviors, and personality disorders as significant emotional factors in autism severity (29). The association between mild autism and self-isolation resonates with recent research emphasizing the intricate risk profile associated with dual isolation behaviors. It also correlates with a study on an emotional sample, linking self-isolation as a primary predictor of mild autism in psychiatric children (30).

In conclusion, it is evident that the SSRS, with or without the supplementary assessment of the NEGP, exhibited improved treatment accuracy in the studied population compared to the majority of previous studies involving autistic students. This diversity in performance can be attributed to various factors. Firstly, within the limited literature on this subject, relatively few studies have evaluated the recent accuracy of the SSRS, as utilized in the current study. Previous studies often had small sample sizes and brief follow-up periods, which contributed to the observed differences. Additionally, the specific diagnostic focus on autism embraced by our study may have influenced the outcomes.

### 4.4 Treatment accuracy of the SSRS and NEGP

In evaluating the treatment accuracy of the SSRS and NEGP, our study compared results with a parallel study conducted in the same clinical setting and treatment period, involving a cohort of 99 children diagnosed with autism, leading to significantly divergent findings (9, 31). These outcomes echo previous research indicating that while the SSRS showed moderate effect sizes in predicting severe autism in the aforementioned study, the incorporation of the NEGP did not yield significant results. Notably, no substantial correlations between NEGP items and autistic outcomes (such as autistic index isolation, mild autism, severe autism) were discerned. This suggests that the performance of both the SSRS and NEGP, at both item and total score levels, is influenced by the psychiatric diagnoses of autistic students, with a more pronounced efficacy observed in severe autism cases compared to mild autism. Nevertheless, given the larger sample size in our study, the hypothesis warrants further exploration to validate in subsequent research endeavors.

In the realm of clinical implications, our findings buttress the use of the SSRS for evaluating children with autism (32). However, caution is advised when generalizing these results to diverse psychiatric populations, underlining the pertinence of considering the diagnostic context while employing emotional assessment tools (33, 34). Additionally, the therapeutic insights gleaned from the emotional risk factors identified by the SSRS could be invaluable, particularly in scenarios marked by time constraints or limited data availability (35). Although the NEGP did not enhance the treatment precision of the SSRS, it may still hold clinical value in supporting emotion-informed interventions and treatment planning, aiding in cultivating a holistic understanding of the emotional profiles of children with autism and facilitating the customization of treatment strategies (24, 36, 37). Given its swift administration, addressing emotional needs could serve as a beneficial adjunct to autism care. In conclusion, the study underscores the imperative for further research to delve into how emotional assessment instruments operate divergently across various autism diagnoses, underscoring the necessity of attaining a nuanced comprehension of these tools in multifaceted clinical settings. Therefore, future research should prioritize investigating personification abuse and emotional treatment approaches.

### 4.5 Limitations

This study has several notable limitations that warrant acknowledgment. Firstly, the dependence on retrospective record data, which was not specifically collected for this study but rather obtained from existing emotional treatment documentation, resulted in variations in the comprehensiveness and content of the records of children with autism. Additionally, the onset demonstration and observation in this research were approached from a multifaceted perspective. On one hand, authors lacked precise regulations regarding the rating scales used during data collection, such as SSRS, which could potentially mitigate the risks of emotional treatment bias or errors (38). On the other hand, authors should incorporate more statistical data collection procedures to enhance the objectivity and quantifiability of the methodology. To address this issue, future research could benefit from adopting a mixed-methods approach. Moreover, the assessment of severe autism was confined to official records of autism, and more robust conclusions could have been drawn if self-report data or alternative information sources were included (35, 39). Nevertheless, despite these limitations, the study makes a significant contribution to the literature on autism assessment for emotionally-involved children with autism by utilizing a substantial sample size and encompassing all children with autism who underwent emotional treatment at a single psychiatric facility.

## 5 Conclusion

The current study sheds light on significant insights regarding the emotional treatment of autism in children. These findings contribute significantly to autism research by emphasizing the effectiveness of the SSRS in this population, demonstrating high treatment accuracy for both mild and severe autism. Moreover, noteworthy positive effects were seen when the SSRS was combined with the NEGP to address both forms of autism. It is intriguing that integrating the NEGP did not enhance treatment accuracy; however, specific emotion-responsive factors within the NEGP emerged as significant predictors of autistic outcomes (40). Four distinct groups displayed variations in their perceptual force and psychomotor forces while exhibiting relatively consistent performance in static and dynamic patterns. Language disorders and emotional behaviors were identified as significant predictors for both types of autism, with self-isolation behavior associated with mild autism and problematic intimate relationships linked to severe autism. Therefore, this study underscores the appropriateness of the SSRS for emotionally engaged children with autism and highlights the clinical significance of the NEGP. Consequently, the previous findings, in conjunction with the current study’s results, provide insights for future interventions aimed at fostering specific parent-child interaction behaviors to address issues related to personification and abuse.

## Data availability statement

The original contributions presented in the study are included in the article/supplementary materials. Further inquiries can be directed to the corresponding author.

## Ethics statement

The studies involving human participants were reviewed and approved by Human Research Ethics Committees affiliated by Taizhou University. All the participants and caregivers provided written informed consent to participate in this study. Written informed consent was obtained from all the participants and caregivers for the publication of any potentially identifiable images or data included in this article.

## Author contributions

YK: Conceptualization, Formal analysis, Funding acquisition, Methodology, Validation, Visualization, Writing – original draft, Writing – review & editing. XZ: Conceptualization, Data curation, Formal analysis, Investigation, Visualization, Writing – review & editing.
